# Watch out for malaria: still a leading cause of child death worldwide

**DOI:** 10.1186/1824-7288-36-58

**Published:** 2010-09-09

**Authors:** Danilo Buonsenso, Luigi Cataldi

**Affiliations:** 1 Department of Pediatrics, Catholic University of the Sacred Heart, L.go A. Gemelli, 8, 00168, Rome, Italy

## Abstract

**Background:**

Due to the efforts in malaria control promoted by the World Health Organization (WHO), the reported malaria burden is being reduced throughout the world. Nevertheless, malaria remains a leading cause of child death worldwide.

**Aims:**

purpose of the paper is to summarize the main historical steps in fighting malaria, from the first descriptions to the last ones.

**Results:**

a case of probable autochthonous malaria has been recently described in Italy, raising concern over the possibility of resurgence of malaria in countries previously interested by this disease. Moreover, both the constant threat of the parasite and vector mosquito developing resistance to medicines and insecticides, and the on-going climate change make the challenge of eradicating malaria really difficult. Therefore, malaria is still an actual disease, requiring adequate programs of surveillance, stronger health systems in poor countries, and efforts in order to develop new and effective tools in malaria control. WHO has definitely demonstrated the effects of "social determinants" on health. So, eradication strategies cannot be based only on a scientific background, because culture, politics, power, resources and wars have a profound impact on health and disease. These elements should be introduced in all the programs of malaria control.

**Conclusions:**

malaria is still an actual disease with great public health implications, and the approaches for control and prevention should have the appropriate social and political context in addition to the science involved in order to save lives of children at risk.

## Background

Malaria is a protozoal disease, transmitted by the Anopheles species mosquito carrying the *Plasmodium *parasite. It is the most important protozoal disease to affect man, with transmission, until today, documented in 103 countries, affecting more than 500 million people per year, causing between 1 and 3 million deaths, especially children under 5 years, pregnant women and not immune people. Today, malaria is common, above all, in the tropical and sub tropical areas of the world. In some areas of Italy, as in the majority of Mediterranean countries, malaria has beenknown for centuries and the prevalence was reduced only since 1947 [1].

At the beginning of the 20^th ^century, malaria was the biggest problem facing Italian public health. It caused low productivity, poverty and economy underdevelopment. However, Italy became one of the most important world centre for the development of the study of malaria and it was the first country to launch a national campaign to combat the disease. The defeat of malaria was linked with the extension of civil liberties, the spread of education and improvements in the general health of population [2].

Since the end of 1800, in Italy every year there had been 15,000 deaths from malaria, above all in the South. Nevertheless, in Italy has been recently reported a case of malaria, interesting a 44 year old man who had not travelled out of Italy and probably contracted malaria in the Agro Pontino, a former malaria endemic area in Italy [3].

## Methods

Bibliografic research on both Italian and not-Italian books of history of medicine and on MEDLINE/Pubmed.

The aim of this paper is to summarize the main historical and upcoming steps that shaped the complex relationship between man and the malaria parasite.

Table 1 summarizes the main steps about the history of malaria.

**Table 1:** Main steps about the history of malaria.

Historical period	Events
**Ancient history**	
- **≈ 1570 BC**	Ebers *Papyrus*: description of symptoms (fever and spleen enlargement);
- **≈ 1000 BC**	Atharvaveda: description of symptoms and recurrence of the fever;
- **≈ 460 - 370 BC**	Hyppokrates of Kos in his work *Epidemie *describes the intermittent fever;
- **340 AD**	Ge Hong, China, describes the anti-fever properties of *qing hao*.

**17^th ^century**	First description of Peruvian bark (Cinchona), from which quinine is extracted.

**1880**	Alphonse Laveran: the discovery of the Malaria parasite.

**1885 - 1889**	Camillo Golgi: the differentiation of different form of Malaria.

**1897 - 1898**	Ronald Ross: the *Plasmodium *is transmitted by "a mosquito".

**1898 - 1899**	Giovan Battista Grassi: the *Plasmodium *life cycle.

**1905 - 1910**	The control of malaria and yellow fever make possible the construction of the Panama Canal.

**1914 - 1942**	The U.S. Public Health Services and the U.S. Tennessee Valley Authority: the integration of Malaria control with the U.S. economic development

**1934 - 1946**	A new antimalarial drug: chloroquine.

**19^th ^- 20^th ^century**	
- **1874**	Othmar Zeidler describes the synthesis of DDT;
- **1939**	Muller demonstrates the insecticidal property of DDT

**1955 - 1978**	WHO Malaria Eradication Efforts: an unsuccessful "global" campaign with the exclusion of sub-Saharan Africa.

**21^st ^century**	The knowledge of genetic map and the research for an effective vaccine.

## Results

### Main historical steps

It is likely that the history of malaria closely follows the history of man. It is widely held that the parasite was present in the ancestors of the *Homo Sapiens*. Seasonal or intermittent fevers are well documented in ancient civilizations such as the Sumerian, the Assyrian-Babylonian, the Egyptian, the Indian and the Chinese. From these civilizations different written testimonies have survived. The Ebers Papyrus (about 1570 BC) describes the symptoms of the disease, making reference to the enlargement of the spleen accompanied by fever. The Atharvaveda (an ancient Indian text dated about 1000 BC) describes the symptoms and recurrence of the malaria fever. The first detailed clinical description of malaria, however, fell to Hyppokrates of Kos, who in the aphorisms of his work *Epidemie *described the typical intermittent fever.

The origins of malaria remained unknown for centuries. It was thought that miasmas or "bad air" above stagnant water caused the disease. Indeed, the etymology of the word "malaria" (bad-air) stems from the conviction that the disease was caused by miasmas from marshy areas.

In the sixteenth century a different theory regarding the cause of the disease emerged. In 1546, Girolamo Fracastoro (about 1483-1553) from Verona, who studied and was a lector in Padova, as well as being a doctor on the Council of Trent, published *De Contagiose et Contagiosis Morbis*.

In this work he argued that the "contagion" was the mechanism for the transmission of the disease. The rules for human quarantine were subsequently based on this theory [4].

In 1880 in Constantine, Algeria, Alphonse Laveran identified the protozoan for the first time in peripheral human blood. In Rome Ettore Marchiafava and Angelo Celli studied it and named it *Plasmodium*. In Pavia, in 1885, Camillo Golgi (Nobel Prize winner, 1906), demonstrated the association between the recurrence of the malaria fever and the life cycle of the *plasmodium*. He also demonstrated that the two types of malaria fever, the tertiary and the quaternary, were caused by two different species of *plasmodium*: the *P. vivax *was responsible for the tertiary benign type and the *P. malariae *for the quaternary. In 1889 he demonstrated that the fever attacks begin after the first round of blood schizogony. In Rome, Ettore Marchiafava, Angelo Celli, Amico Bignami and Giuseppe Bastianelli demonstrated the existence of the *P. falciparum*, responsible for the tertiary malignant type of malaria.

In China in 1894, Patrick Manson hypothesized that which in India in 1897 Ronald Ross (Nobel prize winner, 1902) demonstrated: the *Plasmodium *was transmitted to man by "a mosquito".

In Rome in 1898, Giovanni Battista Grassi identified the *Anopheles *species as the mosquito carrier of malaria and managed to carry out the first ever experimental transmission. In 1899 he described the complete life cycle of the various species of the *Plasmodium*. Grassi had observed that in places where malaria was rife, there were always mosquitoes. However, not always where there were mosquitoes there was malaria. As a result he hypothesized that only a few species of mosquito could transmit the disease. He spent his summer holidays searching for the suspect "monster mosquito" that appeared only at dusk and had four small light brown narks on its wings, and a raised abdomen: the *Anopheles Claviger*. Grassi entered the homes of those sicks with malaria and asked them about the bites they had received and their habits. He catalogued around 40 species present in Italy. On the 28^th ^of September 1898, at the Lincei Academy, Grassi read a communication in which he assured his audience that only the *Anopheles *mosquito and two other species were suspected of transmitting the disease. That autumn Grassi allowed a friend, Mr Sola, to be bitten numerous times by the *Anopheles *mosquito. Mr Sola was infected and his blood resulted positive for the presence of malarial parasites. After Mr Sola, the patients of an entire ward of the Santo Spirito Hospital (Rome) were bitten by mosquitoes deliberately freed; they were all infected and all came down with malaria. Grassi demonstrated that only the *Anopheles *mosquito was responsible for the transmission of malaria from man to man and that bird malaria could not be transmitted by the same mosquito responsible for human malaria. He taught people in infected areas not to go out at dusk but to stay in their homes and prevent mosquitoes from entering.

In this same period, new research brought along the discovery of a product which would come to represent a cornerstone in the fight against malaria. In 1874, an Austrian student, Othmar Zeidler, while defending his doctoral thesis at the University of Strasbourg, described the synthesis of DDT created by allowing chlorobenzene and chloral to react together. The results of his research were published in 1874. In 1939, Muller demonstrated the insecticidal activity of DDT. In 1940, the compound was patented and in Switzerland in 1941 the first products containing DDT were sold.

The first campaign using DDT began in Naples in 1944, in order to tackle a typhus epidemic. Over 3 million individuals, both civilian and military were "dusted" with the mixture of insecticide. DDT began to develop its reputation as the "miracle insecticide". It then showed its effectiveness against mosquitoes carrying malaria in the Pacific. From 1947 to 1952, areas with malaria in Italy were subjected to treatment with DDT, which helped to weaken the disease. In the world, the World Health Organization (WHO) estimated that in the first 8 years of use, DDT prevented at least 100 million cases of malaria and 5 million deaths. In 1948, Muller was awarded the Nobel Prize for Medicine for the discovery of the strong insecticidal action of DDT. In 1955, the WHO launched a "global" campaign to eradicate malaria in the world, based on the use of DDT, even though most countries in the sub-Saharan Africa (the area most afflicted by the disease) were completely excluded from the campaign efforts. This brought about a weakening of the disease in developed countries and in many areas of Asia and Latin America. In 1969, the global campaign was interrupted by difficulties, above all, in areas with a high incident rate of the disease (Sub-Saharan Africa). During the campaign to control malaria, DDT showed a lot of side effects, both on mankind and environment. Indeed, in 2001 the Stockholm Convention gave permission for the production and use of DDT for the control of vectors of diseases, in particular malaria. However, conditions set out in the treaty would have to be followed and its use could only occur after specific recommendation from the WHO [5].

At the beginning of 2000, the knowledge of the genetic map of the malaria parasite gave hope that new action plans could be developed as well as new therapeutic targets and plans for the development of a vaccine [6][7].

### Treatment

Today therapeutic strategies are based on an "intercultural collaboration", which relies on the use of mosquito nets, DDT and multi-drug cocktails. Different schools of thought are united by the common and ambitious objective: to weaken one of the most insidious and long-lived diseases in the history of mankind.

Medical treatment dates back to antiquity when each attempt was based on relative theories of how and where the disease originally developed. Such theories relied on people's observations of the disease and on what they believed were the truths about the universe, life, illness and god.

Ronald Ross, the first to find the malaria parasite and later awarded the Nobel Prize, believed in attacking the carrier, by blocking mosquitoes and protecting oneself with a net and not living near the miasmas. He was a firm believer in the theory of miasmas and used it as a base for his fight to control malaria. He was convinced that creatures living in the "miasmatic" environment were responsible for the transmission of malaria.

Robert Koch believed in the necessity of preventative treatment with quinine for anyone in an area hit by malaria. His approach was to "treat the patient not the mosquito". This was a form of drug induced quarantine for a healthy patient, based on the belief that malaria was a "germ" passed from person to person.

The control of malaria has drawn from both paradigms. Naturally miasmas are part of and have effects for the entire community. Ross wanted to eliminate them. The Italians did it in the Agro Pontino with Mussolini (a particularly shocking aspect to the history of this area is mentioned in Snowden's book [2] and describes how the retreating Nazi armies in Italy in 1943-44 deliberately caused a massive malaria epidemic in Lazio. It was "the only known example of biological warfare in 20^th^-century Europe"). They drained the marshland between Rome and the sea, which drastically reduced the spread of malaria in that area. Koch recommended the widespread use of quinine to colonial governments. The program for the eradication of malaria of the WHO also focused on the use of DDT, even though only after specific recommendation and in selected cases [8][9].

Artemisinine is the endoperoxide of a sesquiterpene lactone derived from the infesting plant *qing hao*, also known as sweet or annual wormwood. The Chinese attributed medical properties to this plant more than 2000 years ago. In 340 AD, Ge Hong prescribed tea of *qing hao *as a remedy for feverish attacks and in 1596, Li Shizhen recommended it for the relief of the symptoms of malaria.

In 1972, Chinese researchers extracted and crystallized the principle anti-malarial component, *qinghaosu*, now known as artemisinin. Artemisinin has now been replaced by three more powerful semi synthetic derivatives, which are more widely available: dihydroartemisinin, artemether and artesunate.

Quinine is the main alkaloid extracted from the powdered bark from the *quina *plant (Cinchona) from South America, also known as Peruvian, Jesuit or cardinal bark. The name of Cinchona for the "fever tree" came into existence as a result of the successful treatment of the countess of Quinhon, wife of the Spanish Vice-king of Peru. She was suffering from malaria and was given the indigenous medicine, bark of the "fever tree". It was used by the indigenous Peruvians as a cure for the shivers. In 1633 an Augustinian monk called Calancha from Lima, Peru, was the first to report that the powder of the cinchona plant "given as a drink cures fever and tertian". In 1640, cinchona was used to cure fever in Europe. The Jesuit Fathers were the main importers of cinchona in Europe. For almost two centuries the bark of the cinchona tree was used in medicine as a powder. In 1820 Pelletier and Caventou isolated quinine from cinchona bark. Quinine is still today fundamental to the cure of attacks of malaria from *P. falciparum *resistant or multi-resistant to chloroquine.

Chloroquine has been valued during a collaborative program of research into anti-malarial drugs during the Second World War. Starting in 1943, thousands of these compounds were synthesized and tested for their anti-malarial activity. Chloroquine showed itself to be the most promising. At the end of the war it was discovered that the same compound had been synthesized and studied under the name of Resochin by the Germans since 1934, but it had been rejected because of the toxicity demonstrated after testing on birds [10].

However, one of the largest present medical emergencies is the ever increasing resistance to drugs used to treat the most important micro-organisms that attack mankind. This has been caused by the large scale and often incongruous use of various anti-microbial drugs. This problem can naturally concern the fight against malaria. Preliminary studies have highlighted this problem. In a randomized study published in the New England Journal of Medicine (NEJM), 94 adults from the Battambang province, in West Cambodia, with non complicated malarial infection from *P. falciparum *were treated with artesunate (4 mg per kg of body weight per day, for 7 days), or quinine (30 mg per kg per day) in addition to tetracicline (25 mg per kg per day) in separate doses every 8 hours for 7 days. Patients were observed for 28 days after the first day of treatment. Four patients had an increase in parasitemia between the 21^st ^and the 28^th ^day, of these, two were classified as resistant to artesiminin, in adherence with the parameters set by the authors of the study. This study showed that artesiminine is still a powerful drug, which reacts very quickly, allowing little time for the parasites to modulate their genes in order alter the targets of the drug. Nevertheless, the widespread use of this drug, with the demonstration in this work of the theoretical possibility of resistance to artesiminine resulting from the long time period required for the clearance of the parasite, makes the discussion over resistance more problematic. It also underlines the importance of the production of a vaccine, not only for these areas but for all of humanity [11].

A recent discovery (2010) has opened new futuristic scenarios about the identification and development of new strategies on how to control the spread of malaria. A study published on *Nature *by Modiano et al ("*La Sapienza*" University, Rome) demonstrated that variants in genes that have been shown to be protective against malaria (hemoglobins C (HbC, beta6Glu-->Lys) and S (beta6Glu-->Val)) were associated with an increase of parasite transmission from the human host to the *anopheles *vector. This is an example of how genetic variation may influence the transmission dynamics of an infectious disease. The host-vector transmission can be affected also by genetics factors [12].

### Vaccine

An effective vaccine for malaria has been the subject of research for over 70 years. Various antigens have been identified as potential targets for the development of an anti-malaria vaccine.

One of these, the repetitive sequence of 4 amino acids of the antigen of the circumsporozoite on the surface of the *Plasmodium falciparum*, forms the basis of the RTS,S vaccine. Initial results on human volunteers indicate an effective protection of 40% when the vaccine is used in combination with an effective adjuvant therapy. This vaccine would have an estimated effectiveness of about 30% in its capacity to reduce episodes of clinically manifested illness, and about 40% in cases of new infection. In the NEJM, Abdulla et al [13] described the trial in which the RTS,S vaccine was used in combination with the adjuvant AS02 D. The result was a reasonable effectiveness when compared to the Hepatitis B vaccine and the strength of anti-circumsporozoite anti-bodies was recoverable in 98% of infants who received the vaccine. In this trial, the vaccine was given together with other vaccines (diphtheria vaccine, tetanus toxoid, whooping cough, *Haemophilus influenzae *type b) as foreseen by the schedule of the Expanded Program on Immunization (EPI). There was no interference with the other vaccines. This led scientists to believe that it could be administered together with other vaccines in areas where malaria is endemic. During the six months of observation following immunization, the incidence of malaria infection and clinical illness of the group RTS,S was reduced by 65% and 59% respectively. There was a correlation between the reduction of the risk of infection and the increase in the strength of circumsporozoite anti-bodies but there was no association between the reduction of the incidence of clinically manifested malaria and the strength of antigens. Bejon et al [14] published on the NEJM the results of a trial of phase 2b regarding the effectiveness and safety of the RTS,S vaccine when combined with the ASOIE adjuvant for children aged between 5 and 17 months. The vaccine was associated with less collateral effects when compared to the anti-rabies vaccine. It had a 60% effectiveness against episodes of malaria clinically manifested from the *P. falciparum*, with anticircumsporozoite antibodies present in 99% of those vaccinated. Even in this case there was no correlation between the protection against clinically active malaria and antibody strength. In this vaccine the antibody strength was about 10 times superior to Abdulla's vaccine, but the effectiveness is analogous. It is not demonstrated whether the higher antibody strength is associated with a longer duration of effective protection. Acquired immunity against one form of malaria parasite, does not mean protection against the other forms. The target of the RTS,S vaccine is the sporozoite. Therefore, if the immunity acquired by the vaccine fails to block even one sporozoite, this can lead to the hepatotropic stage and from there to the erythrocyte phase and then to illness. Even though the first results are promising, the incidence rate of malaria in the studied areas was low. In addition, the evaluation of the vaccine has been complicated by the widespread use of insecticides and treatment programs based on the use of artemisinine. For example, in areas where malaria is endemic, such as Gambia in western Africa, and in Kenya and Tanzania in the east, there has already been an important reduction in the force of malaria. These data make statistical interpretation of the obtained results more difficult. In 2009, the RTS,S vaccine will begin phase 3 of the trial, even in areas where transmission of malaria is moderately high. This is the first anti-malaria vaccine to reach phase 3 and the forthcoming studies in areas with high transmission levels will allow scientists to understand the true value of this vaccine. Even though the vaccine against malaria is still at a very early stage of development and nobody knows at this time whether a sufficiently effective and affordable vaccine against malaria will ever see the light, it is a promising start [15].

### Malaria and cancer

Malaria is not only an infectious disease: the relationship between endemic *Plasmodium falciparum *malaria and *Epstein-Barr *virus (EBV) infection in the genesis of endemic Burkitt's lymphoma (eBL) is well established. eBL, the most prevalent childhood cancer in equatorial Africa, is a high-grade B cell lymphoma characterized by a c-myc translocation and the consistent presence of EBV. After primary infection, EBV establishes a lifelong persistent infection. African children are infected by EBV early in life while seroconversion tends to occur later in developed countries.

Acute and chronic malaria infections profoundly affect the B cell compartment, inducing polyclonal activation, hyper-gammaglobulinemia and a dramatic increase in the levels of circulating EBV. A recent study showed data suggesting a molecular link between the parasite, the B cell and EBV: the cystein-rich inter-domain region 1 (CIDR1) of the *Plasmodium falciparum *erythrocyte membrane protein 1 (PfEMP1) seems to function as a polyclonal B cell activator. CIDR1 increases B cell survival and activates the memory compartment where EBV is known to persist. Moreover, *P. falciparum *antigens such as PfEMP1 can directly induce EBV reactivation during malaria infections. In conclusion, the increased viral load and the concomitant polyclonal B cell activation with enhanced B cell survival may augment the risk of eBL development in children living in malaria-endemic areas [16][17].

### Malaria and pregnancy

Malaria infection during pregnancy is one of the contributors to neonatal mortality, mostly through low birth weight (LBW) and by causing maternal anemia [18].

In malaria stable transmission areas in Africa, approximately 25 million pregnancies are exposed every year to the infection. In these areas, WHO recommends preventive strategies during pregnancy [19][20].

These measures are based on the administration of intermittent preventive treatment (IPTp) and the use of insecticide treated nets (ITNs).

IPTp is the administration of two or more treatment doses of an effective antimalarial drug given at defined intervals during pregnancy. The currently recommended regimen for IPTp is at least 2 treatment courses of sulphadoxine-pyrimethamine (SP) given from the 2^nd ^trimester onwards at least one month apart. It acts as treatment of existing infections (symptomatic or not) and as prophylaxis by limiting the development of infection for an interval of time in pregnancy [21].

In a recently published trial, it was found that IPTp with SP was associated with a moderate but significant reduction in the incidence of clinical malaria during pregnancy, and with a statistically significant reduction in the prevalence of parasitaemia at delivery and at 8 weeks postpartum, as well as in fetal anemia in cord blood [22].

However, the uptake of these tools varies between countries but it is still far from covering the majority of the pregnant women at risk of malaria in Africa [23].

Nevertheless, it is not yet well established if these strategies are associated with a minor prevalence of low birth weight (LBW), prematurity or maternal anemia at delivery [21][24].

On the other hand, a recent work shows that IPTp in pregnancy in regions with high malaria transmission and high drug resistance may increase the overgrowth of resistant parasites and worsen malaria infections [25].

In malaria-endemic areas, pregnancy increases susceptibility to infections by the malaria parasite *Plasmodium falciparum*. Pregnant women with *P. falciparum *malaria are more likely to have LBW babies and to suffer from anaemia, especially during their first pregnancy.

Identifying the determinants of immunity to malaria in pregnancy is critical to understanding the pathogenesis of the disease, and having a reliable and convenient measure of protection from infection in pregnant women who live in endemic regions is an important goal for local and international public health authorities [26].

## Discussion

More than one hundred years have passed since Laveran, Ross and Grassi discovered the details of malaria transmission. Nevertheless, malaria is still an actual disease with great public health implications: still today more than 500 million people in the world contract malaria every year and 1 million people die, overall children.

Malaria is an actual but above all potential concern, because of some elements that could further affect its epidemiology and treatment:

1. Climate changes could recreate favorable environmental conditions may increase the spread of the disease in areas that already experience serious problems. Scientists have developed models to predict the effects of climate change on malaria spread in the next decades. Many of these models predict an expansion in the transmission of the disease in the next thirty years. The data provided by the MARA project (Mapping Malaria Risk in Africa) predict that, if the population does not increase, in 2100 in Africa the percentage of people exposed to malaria will be 16-28% more than today [27].

2. Treatments nowadays available may no longer be as effective as they presently are because of increasing threat of drug resistance.

3. The administration of anti-malarial drugs is no doubt more difficult in countries where the socio-cultural context is different from developed countries one. The introduction of an effective vaccine could certainly be a solution to this problem because of its easier way of administration. Moreover, it could represent a "problem" for its wide distribution in poor countries the fact that western market will regulate the access to an eventual vaccine.

4. The approaches to prevent and control diseases have their roots in science, but not only; the control programs for the disease have a social and political context that is not merely scientific. Our science is based on a historic-political-cultural-social context different from that of countries where malaria burden is higher. Our context is a "Northern one", with its roots in Western civilization. But nowadays malaria is in the "South", in tropical countries of the Third World, in Latin America, in Africa, in South-East Asia, places not rooted in the Western context. Actual programs for the control of malaria could therefore contrast with the indigenous contexts [8].

An important step forward would therefore be to think of a way of controlling malaria in agreement with all these elements. Motivation of populations and governments and the firm will and ability to establish effective health care systems are a prerequisite for undertaking malaria control.

## Competing interests

The authors declare that they have no competing interests.

## Authors' contributions

DB and LC have made substantial contributions to conception of the article, acquisition of data and their analysis and interpretation. DB have been involved in drafting the manuscript. LC have been involved in revising it critically for important intellectual content and in giving final approval of the version to be published. All authors read and approved the final manuscript.
